# Zinc oxide nanoparticle chelated phosphocreatine-grafted chitosan composite hydrogels for enhancing osteogenesis and angiogenesis in bone regeneration

**DOI:** 10.3389/fmed.2025.1729401

**Published:** 2025-12-05

**Authors:** Leidong Lian, Dingli Xu, Chaonan He, Zhe Luo, Han Yu, Botao Liu, Ke Zhou, Liangjie Lu, Kaifeng Gan

**Affiliations:** 1 The Affiliated Lihuili Hospital of Ningbo University, Ningbo, Zhejiang, China; 2 Health Science Center, Ningbo University, Ningbo, China; 3 Ningbo Institute of Innovation for Combined Medicine and Engineering, The Affiliated Lihuili Hospital of Ningbo University, Ningbo, Zhejiang, China

**Keywords:** bone regeneration, osteogenesis, phosphate-functionalized, chitosan, zinc oxide nanoparticles

## Abstract

**Introduction:**

The natural polysaccharide-based injectable hydrogels have showed significant interest to use as 3D scaffolds for critical-sized bone defect repair.

**Methods:**

Here, we incorporated ZnO nanoparticles (NPs) into a newly synthesized water-soluble phosphocreatine-functionalized chitosan (CSMP) water solution to form an injectable hydrogel (CSMP-ZnO) via supramolecular combination between phosphate groups in CSMP and Zinc in ZnO NPs.

**Results:**

The phosphocreatine in this hydrogel not only provides sites to combine with ZnO NPs form supramolecular binding but also serves as the reservoir to control Zn^2+^ release. The results show that the lyophilized CSMP-ZnO hydrogels presented a porous structure with some small holes in the pore wall, as shown by scanning electron microscopy. Rheological characterizations revealed that the mechanical properties of the hydrogels were almost maintained upon the addition of ZnO NPs. *In vitro* experiments showed that the CSMP-ZnO hydrogel exhibits excellent angiogenic and osteogenic properties compared with the CSMP hydrogel. The as-released Zn^2+^ ions promote the high expression of osteoblast collagen 1 proteins and accelerate bone mineralization by activating the BMP2/SMAD signaling pathway. *In vivo*, the as-released Zn^2+^ ions promot osteoblastic proliferation and the mineralization of osteoblasts inside the CSMP-ZnO scaffolds. Immunofluorescence for RUNX2, COL-1, and CD31, showed that stable vasculature could be formed inside the CSMP-ZnO scaffolds.

**Discussion:**

Both the *in vitro* and *in vivo* results demonstrate that CSMP-ZnO hydrogel shows promise for bone regeneration, suggesting a new strategy for tissue engineering and regeneration in the future.

## 1 Introduction

Critical-sized bone defects are usually caused by genes, infection, trauma, osteomyelitis surgery, or tumors (1, 2). Conventional treatment methods for critical-sized bone defects include autografts, allografts, xenografts, and synthetic bone grafts. However, this method is constrained by the shortage of autologous grafts, complications at the donor site, immune rejection, and high surgical risks (3–5). To address these challenges, researchers have focused on newer materials,which can produce better results, reduce costs, and overcome problems associated with existing grafts. Bone regenerative medicine has been viewed as a promising alternative to conventional bone grafts (6, 7). Over the past decades, various approaches for bone regeneration have been studied, including tissue engineering, biomaterials, and stem cells (8–10). Among the synthetic biomaterials, hydrogels comprising natural or synthetic polymers, which exhibit excellent mechanical properties and biocompatibility, are ideal scaffolds to emulate the extracellular materials for cell proliferation and differentiation (11, 12), and natural polymers such as polypeptides, polysaccharides, and polynucleotides have been utilized to develop hydrogels (13, 14). Despite the progress in developing hydrogels, appropriately incorporating therapeutics into hydrogels to efficiently promote bone regeneration is still challenging (15–17).

Chitosan (CS) is a natural polysaccharide derived from crustacean shells (18). CS has been widely used in bone tissue engineering due to its high biocompatibility, biodegradability, biosafety, bioactivity, antibacterial activity, nontoxicity, antimicrobial, and other properties (19). The structural similarity of CS to glycosaminoglycans, the major component of the extracellular matrix of bone, has made it a suitable scaffold material for tissue engineering. These properties make CS a promising biopolymer for various applications in the biomedical field (20). CS contains a significant amount of hydroxyl and amino groups in its molecular chain structure, making it highly susceptible to the creation of strong intramolecular and intermolecular hydrogen bonding, as well as intermolecular van der Waals forces, thereby forming a more stable and organized crystalline structure, which ultimately leads to its low solubility. Therefore, CS is not soluble in water or alkaline solutions, but in inorganic acids such as dilute hydrochloric acid and dilute nitric acid, or some organic acids. CS contains three reactive functional groups: an amino group at the C2 position, a primary hydroxyl group at C3, and a secondary hydroxyl group at C6. The solubility and functionality of CS can be improved by chemically modifying these functional groups and introducing new ones. Currently, the chemical modifications for CS include acylation, alkylation, sulfonation, esterification, etherification, carboxylation, silylation, and graft copolymerization. These methods can not only retain the original properties of CS but also form CS derivatives with different functionalities, thus expanding the scope of CS application. Integrating this polymer into the structure of various inorganic materials can boost osteoblast cell growth and facilitate bone fracture healing (21–24). One attractive and frequently used modification is the methacrylation of CS, and this CS methacrylate can be used to make the photo-cross-linkable hydrogels. In addition, phosphorylation of CS is also an appealing strategy for bone regeneration applications, as phosphate is one of the main inorganic components in the bone matrix and can chelate metallic ions such as zinc, calcium, and magnesium, thereby promoting the formation of mineral bone (25, 26).

Zinc is an essential micronutrient for the human body and participates in fundamental biological processes, including energy metabolism, enzyme activity, and protein synthesis (27, 28). In the human body, 85% of zinc is in muscles and bones. Bone tissue zinc levels increase as bone mineralization increase. Alkaline phosphatase uses zinc as a co-factor and is involved in bone mineralization (29–32). Zinc can also stimulate osteoblast proliferation and increase the expression of osteoblast gene markers and calcium deposition in the human bone marrow mesenchymal stem cells (33–35). Cho and Kwun found that zinc induces *Runx2* through canonical BMP-2 signaling (36). Recent studies suggest that incorporating zinc into synthetic ploymer scaffolds for bone tissue engineering can enhance osteoblast differentiation and accelerate bone regeneration by Zn ions (37, 38). Zinc oxide nanoparticles (ZnO-NPs) have gained increasing attention for their high biocompatibility, chemical stability, and limited side effects (39). ZnO-NPs are listed as ‘generally recognized as safe material’ by the US Food and Drug Administration which possess excellent antibacterial and osteogenic capabilities (40). Qiu et al. investigated ZnO-NPs-modified CS/borosilicate bioglass composite scaffold for inhibiting bacterial infection and promoting bone regeneration (41). Yi et al. combined ZnO with mineralized collagen scaffolds with osteoinductive properties to stimulate the recruitment and differentiation of stem cells while achieving antimicrobial activity through controlled release of zinc ions, effectively treating infectious bone defects (42). To date, ample evidence suggests that bone implant scaffolds coordinated with ZnO-NPs can enhance osteogenic abilities (43, 44). ZnO NPs also activate vascular endothelial growth factor (VEGF) expression and stimulate cell proliferation, migration, and tube formation for angiogenesis (45).

**Scheme 1:**
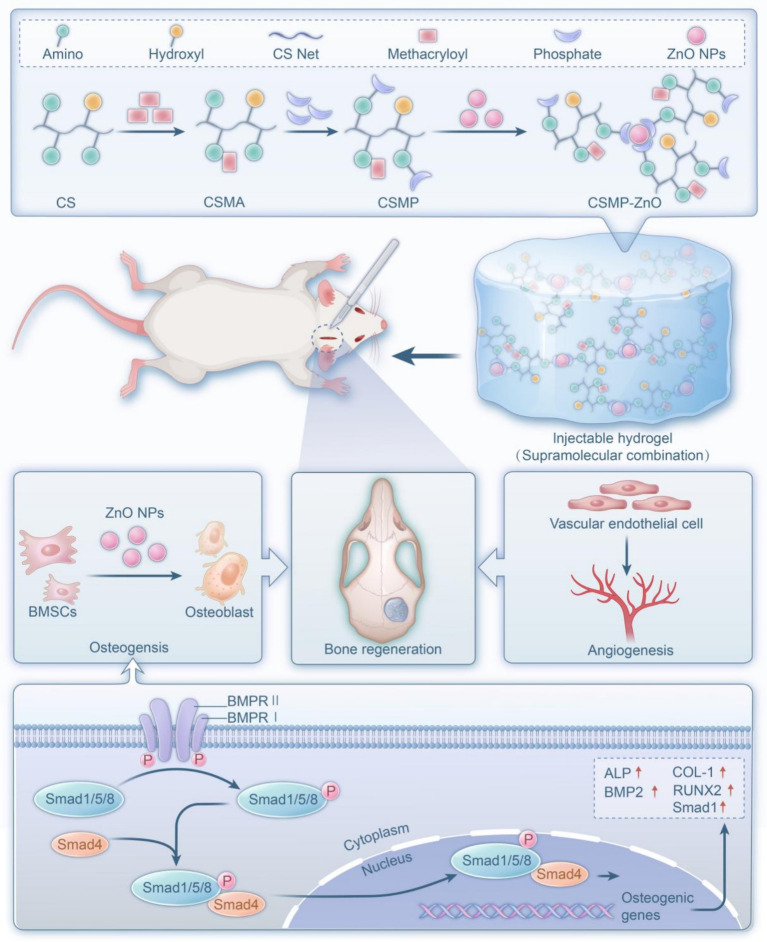
Schematic diagram for the construntion of the CSMP-ZnO hydrogel and its transplantation at rat cranial defects *in situ* bone regeneration.

In this study, the ZnO-NPs were incorporated into the synthesized methacrylate phosphorylation-functionalized CS (CSMP) to form a novel composite hydrogel via supramolecular combination with compatible bone regenerative properties, including biocompatibility, biodegradability, swelling resistance, improved mechanical, osteogenic and angiogenic properties (Scheme 1).

## 2 Materials and methods

### 2.1 Materials

Chitosan (Mw = 1526.500 g/mol, degree of deacetylation = 95%, viscosity = 150 mPa·s), acetate acid, 2-(N-morpholino) ethane sulfonic acid monohydrate solution (MES Solution), ZnO-NPs (Particle size: 50 ± 10 nm), acrylamide, *N*, *N′*-methylenebisacrylamide, 2-hydroxy-4′-(2-hydroxyethoxy)-2-methyl-propiophenone were purchased from Aladdin Biochemical Technology Co., Ltd. (Shanghai, China). Methacrylate (MA), phosphocreatine (PS), *N*-(3-dimethylamino propyl)-*N′*-ethylcarbodiimide hydrochloride, *N*-hydroxysuccinimide were purchased from Sigma-Aldrich, St. Louis, Mo, USA. Rat bone marrow-derived mesenchymal stem cells (rBMSCs) were acquired from Pricella (Wuhan, China), and human umbilical vein endothelial cells (HUVECs) were sourced from Meisen (Zhejiang, China). Dulbecco’s Modified Eagle Medium (DMEM) and penicillin–streptomycin were purchased from Gibco (Thermo Fisher Scientific, USA). Fetal bovine serum (FBS) was obtained from Corning (New York, USA). The Cell Counting Kit-8 (CCK-8) reagent and Alizarin Red S (ARS) staining solution were purchased from Solarbio (Beijing, China).

### 2.2 CSMP synthesis

Three grams of CS were added to 300 mL of 1% acetate acid solution. Then 2 mL methacrylate was slowly added to the CS solution. After stirring at room temperature for 12 h, a CS methacryloyl (CSMA) solution was obtained. Then, 2.4 g phosphocreatine was dissolved in 300 mL of 0.5 M 2-(*N*-morpholino) ethane sulfonic acid monohydrate solution. Then, 4.8 g *N*-(3-dimethylamino propyl)-*N′*-ethylcarbodiimide hydrochloride and 2.4 g of *N*-hydroxysuccinimide were dissolved in the PS solution. Nest, the CSMA solution was added dropwise to the above solution. After reacting at room temperature for 24 h, the solution was dialyzed with deionized water (DIW) water for a week. The dialyzed solution was lyophilized to obtain the flocculent-like CSMP.

### 2.3 Composite hydrogel preparation

The CSMP was dissolved in DIW at a 20 mg/mL concentration. Subsequently, 12.5, 25, and 50 mg of ZnO-NPs were added into 50 μL DIW. The particles were dispersed ultrasonically, and this solution was added to 5 mL of 20 mg/mL CSMP solution with rapid stirring. The concentrations of ZnO in these hydrogels were 2.5, 5, and 10 mg/mL. After mixing, the solutions were left to stand for hydrogel formation. The different concentrations of ZnO in these hydrogels were marked as CSMP-ZnO (2.5, 5, and 10). CSMP was formed using chemical cross-linking. Acrylamide 0.2 g, 2 mg *N*, *N′*-methylenebisacrylamide, and 4 mg 2-hydroxy-4′-(2-hydroxyethoxy)-2-methyl-.

propiophenone were dissolved into the above-prepared CSMP solution, followed by UV irradiation for 30 min to form the CSMP hydrogel. Subsequently, the hydrogels were immersed in DIW for a week to remove any non-reacted reagents from the hydrogel, and the DIW was changed twice daily. The obtained hydrogels were stored in a refrigerator at 4 °C for further experiments.

### 2.4 Characterization

The surface morphology of hydrogels was observed using a scanning elemental microscope (SEM: GeminiSEM 360, Zeiss, jena, Germany). The hydrogels were lyophilized and sprayed with gold. The elemental composition and distribution of the samples were detected through energy dispersion spectroscopy (EDS) in the surface mapping mode, which was equipped with the same SEM device.

The chemical composition of the hydrogels was detected using a Fourier transform infrared spectroscope (FTIR: IRTracer 100, Shimadzu, Kyoto, Japan).

The rheological experiments of the hydrogels were carried out in the oscillatory mode using a rheometer (Kinexus Lab^+^, NETZSCH, Germany). The hydrogels were spread on a parallel plate (25 mm), and a dynamic frequency scan in the range from 0.1 to 100 rad/s was used to record the storage and loss moduli, G′ and G′′. The stress amplitude and temperature were 1% and 25 °C, respectively. Each group underwent three times independent.

Cylindrical hydrogel samples (15 mm in diameter and 10 mm in height) were used for the compression test, which was performed using an electronic universal testing machine (China-UTM-5105). The compression speed was 0.5 mm/min, and the load–displacement curves were transformed into stress–strain curves. The maximum stress was taken from the turning point on the curve, and the compressive modulus was calculated from the linear range in the stress–stain curve before the turning point.

The swelling ratio of the hydrogels was measured by immersing the fabricated cylindrical hydrogels (dry weight, W_0_) in DIW at room temperature. At the set time points, the height of hydrogels was weighted (W_1_) until they were swollen. Each group underwent three times independent. The degradation rate formula for hydrogels is as follows:


$$\text{Swelling ration}\;(100\%)=\frac{W1}{W0}\times 100\%$$

The *in vitro* degradation behavior of the hydrogels was evaluated by soaking the lyophilized hydrogels in phosphate-buffered saline (PBS, Pricella, Wuhan, China) in a static state at 37 °C for up to 28 days, and the PBS solutions were changed every 2 days. In brief, the lyophilized hydrogels (weighted as W_0_) were immersed in PBS at 37 °C. At set time points (days 1, 3, 5, 7, 14, and 21), the hydrogels were lyophilized and weighted as W_1._ Each group underwent three times independent. The degradation rate formula for hydrogels is as follows:


$$\text{Degradation rate}\;(100\%)=\frac{W0-W1}{W0}\times 100\%$$

For zinc ion release, the hydrogel scaffolds were soaked in a centrifuge tube with 5 mL PBS solution. At different time points (days 1, 3, 5, 7, 14, 21, 28). The zinc ion concentration in these collected PBS solutions was detected using inductively coupled plasma atomic emission spectroscopy (ICP-AES, Agilent 5,800, USA). Each group underwent three times independent.

The extraction of CSMP, CSMP-ZnO (2.5), CSMP-ZnO (5), and CSMP-ZnO (10) scaffolds was prepared as previously described by the International Organization for Standardization method (ISO10993-12) (46). Briefly, the scaffolds were incubated in a culture medium for 24 h at a mass/volume ratio of 100 mg/mL at 37 °C. After 24 h, the supernatants were collected and filtered through a sterilized 0.22 mm Millipore (Biosharp, China) and stored at 4 °C (ISO 10993-12) for further use.

### 2.5 Cell culture and cell seeding

Rat bone marrow-derived mesenchymal stem cells (rBMSCs, Pricella, Wuhan, China) and human umbilical vein endothelial cells (HUVECs, Meisen CTCC, Zhejiang, China) were, respectively, cultured in *α*-MEM and endothelial cell medium (ECM) supplemented with 10% fetal bovine serum (FBS, Corning, USA) and 1% penicillin–streptomycin (Gibco, USA) solution in a humidified incubator with 5% CO_2_ at 37 °C. The medium was changed every 2 days. When cell growth reached 80–90% confluence, the cells were digested with trypsin (Gibco 25,200,056, USA). The cell suspension was centrifuged to remove the medium after terminating the digestion by adding the cell medium. The cells were resuspended in a new cell culture medium, and the suspending solution was seeded into a plate for subsequent experiments.

### 2.6 Cell toxicity and proliferation

To study the effect of hydrogels on the proliferation and viability of rBMSCs and HUVECs, 5 × 10^3^ rBMSCs and HUVECs were seeded in a 24-well plate and incubated in the scaffold extraction medium or standard medium (control) for 1, 3, and 5 days. Subsequently, the cell viability was assessed using the cell counting kit-8 (CCK-8, Biosharp, China). The absorbance values at 450 nm were measured using a microplate reader (Thermo Fisher Scientific, USA). Each group underwent three times independent.

Live/dead staining tests were conducted to evaluate the *in vitro* cytotoxicity of the hydrogels. 1 × 10^5^ rBMSCs and HUVECs were seeded in a 24-well plate and incubated in the scaffold extraction medium or standard medium (control) for 1, 3, and 5 days. The cells were immersed in PBS containing 4 mM calcein acetoxymethyl ester (AM) and 16 mM propidium iodide (PI) for 30 min. Dead cells (PI, red) and live cells stained with calcien (AM, green) were observed with a fluorescence microscope (Leica DMi8, Germany).

### 2.7 *In vitro* osteogenic differentiation of BMSCs

#### 2.7.1 Extracellular matrix mineralization assay and alkaline phosphatase (ALP) activity assay

The osteogenic differentiation culture medium was prepared by adding 10 mM *β*-sodium glycerophosphate (Aladdin Chemistry), 50 μM L-ascorbic acid (Aladdin Chemistry), and 10^−7^ M dexamethasone (Aladdin Chemistry) to the standard culture medium. This medium promotes ALP activity and calcium phosphate deposition. About 1 × 10^5^ rBMSCs were seeded on a 24-well plate and then incubated in extractions prepared from osteogenic differentiation culture medium for 14 and 21 days, and the medium was changed every 2 days. Next, the cells were fixed with 4% paraformaldehyde (Solarbio, Beijing, China). Then, the samples were washed thrice with PBS for 10 min each time. The samples were stained using an ALP staining kit (Beyotime, Shanghai, China) and an Alizarin Red staining kit (ARS, Solarbio). On days 14, ALP activity was measured using ALP activity assay kit (Elabscience, Wuhan, China). Each group underwent three times independent.

#### 2.7.2 Quantitative real time-polymerase chain reaction (qRT-PCR)

4 × 10^5^ rBMSCs were seeded in a 6-well plate for 24 h and grouped into control, CSMP, and CSMP-ZnO groups. These samples were then incubated in the extracts prepared from the osteogenic differentiation culture medium for 7 days. Following that, the extraction solution of the scaffold material was changed every 2 days. Total RNA for each sample was extracted according to the protocol of the RNA extraction kit (TransGen, Beijing, China). The extracted RNA was then reverse-transcribed to complementary DNA (cDNA) with the RT reagent kit (PrimeScript™ RT Master Mix, TransGen), and the cDNA was used for qRT-PCR assay (Thermo Fisher Scientific, QuantStudio 5, USA). The mRNA levels of osteogenic genes recombinant bone morphogenetic protein 2 (*BMP2*), recombinant runt related transcription factor 2 (*RUNX2*), collagen type-I (*COL-1*), SMAD family member 1 (*Smad1*), and *ALP* were evaluated and were normalized with the internal control glyceraldehyde-3-phosphate dehydrogenase (GAPDH). Each group underwent three times independent. The PCR primers were designed to amplify the interest genes, as shown in Table 1.

**Table 1:** PCR primers designed to amplify the genes of interest.

Gene	Primer sequences (5′-3′)
COL-1	F:5′- GCGTAGCCTACATGGACCAA -3’
	R:5′- AAGTTCCGGTGTGACTCGTG -3’
RUNX-2	F:5′- TCGGAGAGGTACCAGATGGG -3’
	R:5′- TGAAACTCTTGCCTCGTCCG -3’
BMP-2	F:5′- GACTGCGGTCTCCTAAAGGTCG -3’
	R:5′- CTGGGGAAGCAGCAACACTA -3’
SMAD1	F:5′- GGTGACTGGGAACGGATCG -3’
	R:5′- CCCAGTTAGCACCGGCTC -3’
ALP	F:5′- TGCAGGATCGGAACGTCAAT -3’
	R:5′- GAGTTGGTAAGGCAGGGTCC -3’
VEGF	F:5′- ACAAATGTGAATGCAGACCAAAG -3’
	R:5′- GGAGGCTCCAGGGCATTAGA -3’
FGF2	F:5′- GCGACCCTCACATCAAGCTA -3’
	R:5′- AGCCAGGTAACGGTTAGCAC -3’
GAPDH	F:5′- CCGCATCTTCTTGTGCAGTG -3’
	R:5′- CGATACGGCCAAATCCGTTC -3’

#### 2.7.3 Western blot (WB) analysis

WB was employed to verify the expression of osteoblast differentiation proteins. The osteoblast-associated proteins COL-1, BMP2, RUNX2 expressions were measured. Around 4 × 10^5^ rBMSCs were seeded in a 6-well plate for 24 h and grouped into control, CSMP, and CSMP-ZnO groups. These samples were then incubated in the extracts prepared from the osteogenic differentiation culture medium for 7 days. Following that, the extraction solution of the scaffold material was changed every 2 days. The radioimmunoprecipitation assay (RIPA) lysis buffer solution (Solarbio) was used to soak the cells for 30 min, and the lysed samples were collected in a 1.5 mL microcentrifuge tube. These samples were centrifuged at 12,000 rpm for 15 min, and the supernatants were collected. A bicinchoninic acid protein assay Kit (Solarbio) was used to detect the total protein concentration. The samples were loaded in sodium dodecyl sulfate-polyacrylamide gel electrophoresis (SDS-PAGE) gel for electrophoresis and transferred to polyvinylidene difluoride (PVDF; Solarbio) membranes. After blocking in 5% milk for 1 h, the membranes were incubated with primary antibodies (Supplementary Figure S1) at 4 °C overnight and washed with tris-buffered saline with tween-20 (TBST; Solarbio) three times. These membranes were then incubated with secondary antibodies (Proteintech, USA). Finally, the blotting results were checked by the imaging system (Tanon 5,200, Shanghai, China). Each group underwent three times independent.

#### 2.7.4 Immunofluorescence staining

4 × 10^5^ rBMSCs were seeded in a 6-well plate for 24 h and grouped into control, CSMP, and CSMP-ZnO groups. Then, these samples were incubated in the extracts prepared from the osteogenic differentiation culture medium for 7 days, and the medium was changed every 2 days. The specimens from each group were washed with PBS solution three times and fixed with 4% paraformaldehyde for 20 min, then washed thrice with PBS. Subsequently, after incubation with 0.5% Triton X-100 (Beyotime) in PBS for 20 min, the specimens were blocked in PBS containing 1% bovine serum albumin (BSA; Solarbio) for 30 min. Next, these samples were incubated with the primary antibodies anti-COL1 (1:200, Affinity, USA), anti-ALP (1:200, Affinity, USA) overnight at 4 °C. The secondary antibodies were then conjugated with fluorescein isothiocyanate (FITC) or tetramethylrhodamine isothiocyanate (TRITC) and incubated at 37 °C for 1 h. The nucleus was stained with 4,6-diamino-2-phenyl indole (DAPI). The images were observed and collected with a fluorescence microscope (Leica DMi8, Germany).

### 2.8 *In vitro* angiogenic differentiation of HUVECs

#### 2.8.1 Scratch assay

The effect of scaffolds on HUVECs migration activity was studied using the scratch assay. Briefly, 6 × 10^4^ HUVECs were cultured in a 6-well plate for 12 h to allow the cells to fully cover the bottom of the plate, and the scratch was made with a 200 μL pipette head. Each well was filled 2 mL of sample extracts. After 24 h of co-culture, the healing of the scratch area was observed under an optical microscope (CKX3-SLP, Olympus, Tokyo, Japan). Each group underwent three times independent.

#### 2.8.2 Transwell assay

Transwell plates with a membrane pore size of 8 μm (Corning, NY, USA) were involved to conduct the transwell assay, where each lower chamber was loaded with scaffolds extraction. 4 × 10^5^ HUVECs were seeded into the upper chamber of 24-well transwell plates and allowed for 24 h migration. Cells migrated from the upper chamber to the bottom of each well were thern immersed in 4% paraformaldehyde for 30 min and stained with a 0.5% crystal violet solytion for 30 min. Finally, three radomly selected visual fields and corresponding images were taken and analyzed with an optical microcsope (CKX3-SLP, Olympus, Tokyo, Japan). Each group underwent three times independent.

#### 2.8.3 Tube formation assay

To detect the tube formation ability of the scaffolds, Matrigel (Corning, NewYork, USA) was spread on the bottom of a 24-well plate. Added 4 × 10^5^ HUVECs within 1 mL of sample extracts into each well. After 6 h incubation, tube formation was observed under an optical microscope (CKX3-SLP, Olympus, Tokyo, Japan). Each group underwent three times independent. Further quantitative analysis of the number of joint and total tube length was conducted with ImageJ software.

#### 2.8.4 qRT-PCR assay

HUVECs were seeded at 4 × 10^5^ cells/mL on 6-well plates for 1 day, then the medium was replaced with sample extracts and continue to culture for 7 days. The experiment procedure has been described in 2.6.2. The expression of classic angiogenic related genes, such as vascular endothelial growth factor (*VEGF*), and fibroblast growth factor 2 (*FGF2*) were analyzed. Each group underwent three times independent. The primer sequences are listed in Table 1.

#### 2.8.5 WB assay

HUVECs were seeded at 4 × 10^5^ cells/mL on 6-well plates for 1 day, then the medium was replaced with sample extracts and continue to culture for 7 days. The experiment procedure has been described in 2.6.3. The expression of classic angiogenic related protien, such as vascular endothelial growth factor (*VEGF*), and fibroblast growth factor 2 (*FGF2*) were analyzed (Supplementary Figure S1). Each group underwent three times independent.

### 2.9 *In vivo* animal study

All the animal feeding and surgical procedures complied with the relevant laws and were authorized by the Ethics Committee of Ningbo University. Twelve 6-week-old female Sprague–Dawley (SD) rats were purchased from the Laboratory Animal Center of Ningbo University of China. They were randomly divided into control group, CSMP group, and CSMP-ZnO group, with nine rats in each group. All the animals were anesthetized intraperitoneally with 3% pentobarbital sodium (30 mg/kg). Under sterile conditions, a midline sagittal incision in the scalp was made to expose the parietal bone, and the pericranium was removed by blunt scraping. A critical-size (5 mm in diameter) defect was created below the right side of the crown bone. Bone defects were washed with sterile normal saline, and the hydrogel of each group was implanted into the defect area. The group without any hydrogel scaffold served as the negative control group. After 4- and 8-week implantation periods, these rats were sacrificed with excessive anesthesia. The whole skull was obtained, and the bone defects were investigated using a micro-CT system (Venus® Micro CT VNC-102, Jiangsu, China). New bone volume (BV), new bone volume/tissue volume (BV/TV) ratios, and trabecular bone number (Tb.N) were calculated using appropriate analysis software. Each group underwent three times independent. Then, these samples were fixed in a 4% paraformaldehyde solution for 7 days, after which the tissues were decalcified by being immersed in 10% ethylenediaminetetraacetic acid (EDTA, Codow, China) solution for 4 weeks. Subsequently, the tissues were embedded in paraffin. The cranial cross sections in the central area of the defect were cut at 5 μm for hematoxylin and eosin (H&E), Masson, and immunohistochemical staining for runt related transcription factor 2 (RUNX2, 1:100, AF5186, Affinity), COL-1 (1:100, AF7001, Affinity) and CD31 (1:100, AF6191, Affinity).

### 2.10 Statistical analysis

SPSS 22.0 was used for statistical analysis. The experimental data were expressed as mean ± SD. A one-way ANOVA was used to compare groups, and the LSD method was used for multiple comparisons. *p* < 0.05 was considered statistically significant.

## 3 Results

### 3.1 Rheological properties of the hydrogel

**Figure 1:**
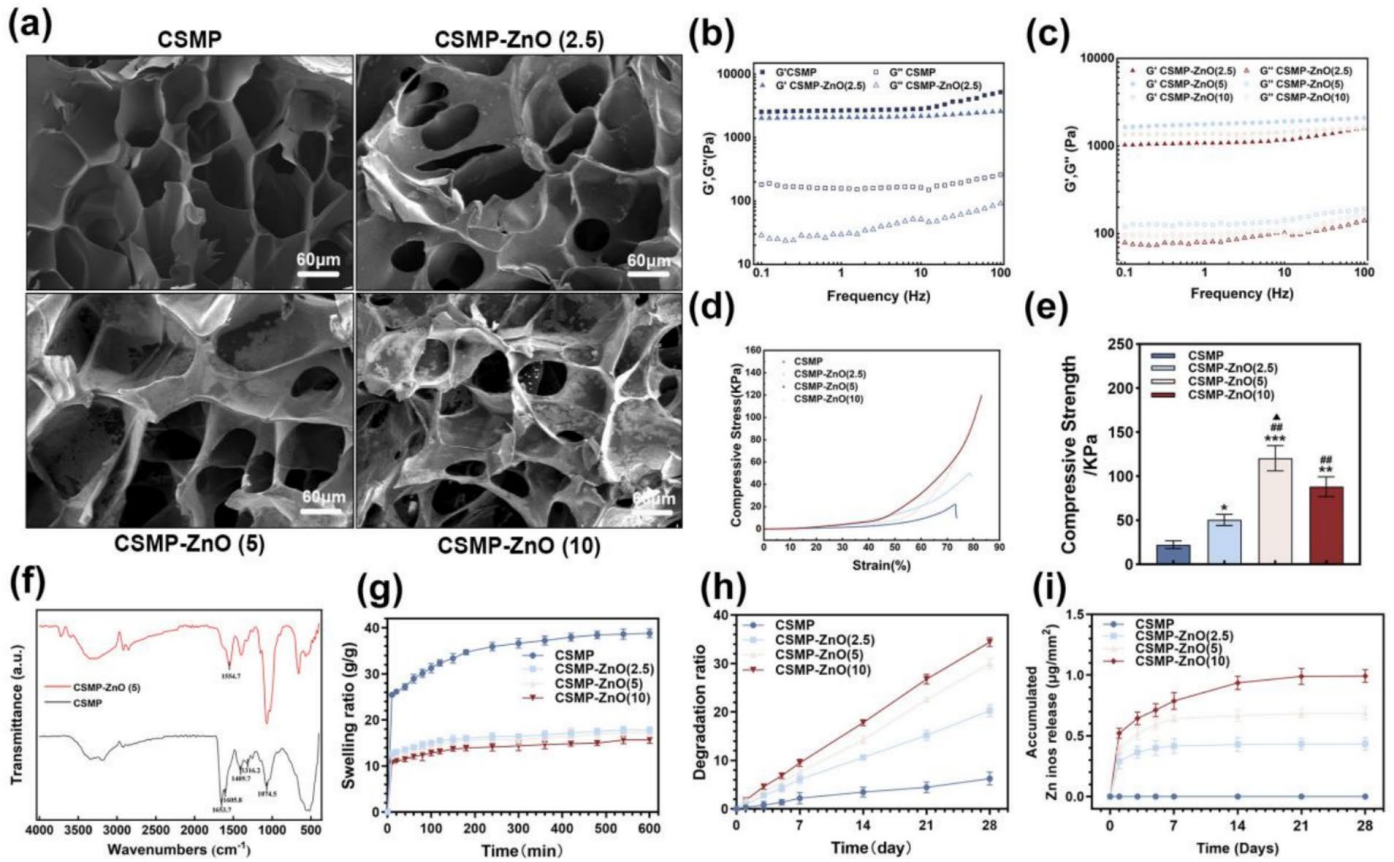
Characterization for hydrogels: **(a)** SEM images; **(b,c)** frequency sweeping of elastic moduli G’ and viscous moduli G” for hydrogels from 0.1 to 100 Hz under 1% stain at 37 °C; **(d)** stress–strain curves; **(e)** maximum compressive strength; **(f)** FTIR spectra; **(g)** swelling ratio of lyophilized hydrogels in DI water as a function of immersion time; **(h)** mass remaining ratio of hydrogels; **(i)** accumulative Zn^2+^ release in the hydrogel scaffolds. Data are presented as mean values ± SD (*n* = 3). *Compared with CSMP. #Compared with CSMP-ZnO(2.5). ▲Compared with CSMP-ZnO (10). *p < 0.05, **p < 0.01, ***p < 0.001; ^#^*p* < 0.05, ^##^*p* < 0.01, ^###^*p* < 0.05; ^▲^*p* < 0.05, ^▲▲^*p* < 0.01, ^▲▲▲^*p* < 0.001.

We carried out rheological experiments to confirm the formation of the hydrogels and characterize their mechanical properties. According to the frequency-dependent oscillatory shear model, the elastic moduli G’ for all hydrogels were 10-fold greater than their corresponding viscous moduli G” (Figures 1b,c), indicating the formation of hydrogels. Upon reaching the plateau, all the hydrogels displayed a storage modulus > 1 kPa, indicating their moderate mechanical characteristics and potential use as scaffolds for bone regeneration. The addition of ZnO-NPs to CSMP hydrogel resulted in a slight decline in mechanical properties, primarily due to the varied ways of hydrogel formations. Among these, CSMP-ZnO (5) had the highest G’ value.

### 3.2 Surface characterization of hydrogels

The surface morphology of lyophilized hydrogels was observed using SEM (Figure 1a; Supplementary Figures S2a,b). All the hydrogels presented a porous structure, and the pore diameter ranged between 50 and 100 μm. The energy-dispersive X-ray spectrometry (EDS) analyses confirmed the homogeneous distribution of C, N, O, P, and Zn elements in the CSMP-ZnO hydrogel (Supplementary Figure S3).

The chemical composition and bonding state of hydrogels were confirmed through FTIR assessments. Based on the FTIR spectra in (Figure 1f), the characteristic peaks of stretching C–N (1316.2 cm^−1^), O–H (1409.7 cm^−1^), N–H bending vibration (1605.8 cm^−1^), and PO_4_^3−^ group (1074.5 cm^−1^) bonds from CS derivates of CSMP were detected for CSMP and CSMP-ZnO (5) hydrogels. Meanwhile, we can observe the C=O (1653.7 cm^−1^) stretching corresponding to the amide bond produced by the CS modification reaction. However, only the characteristic peak for ZnO–P was observed in the CSMP-ZnO (5) sample. The distinctive peak of ZnO–P signifies the combination of ZnO–NPs and phosphate groups in CSMP.

### 3.3 Compressive properties of hydrogels

The compressive stress–strain curves of hydrogels were investigated, and the results are shown in (Figures 1d,e). For maximum compressive strength, CSMP was 22.39 ± 4.33 KPa, CSMP-ZnO (2.5) was 50.55 ± 6.37 KPa, CSMP-ZnO (5) was 120.48 ± 14.24 KPa, and CSMP-ZnO (10) was 88.14 ± 11.24 KPa.

### 3.4 Swelling property of hydrogels

Swelling is an essential characteristic of hydrogels to promote tissue regeneration. The swelling of hydrogels expands the hydrogel volume, weakens their mechanical properties, and oppresses the surrounding tissue, impeding normal metabolism and, consequently, impacting tissue regeneration. Therefore, anti-swelling hydrogels are more desirable for tissue regeneration (47). The swelling property of lyophilized hydrogels was investigated by immersing them in DIW until they swelled (600 min) (Figure 1g), in the initial immersion (0–150 min), the swelling ratio for all the hydrogels was dramatically increased. Over time, the increase in the ratio for all the hydrogels slowed down until they reached the final swollen state. Similarly, CSMP hydrogels showed higher swelling ratios, having the largest swelling ratio, during the entire immersion period compared to CSMP-ZnO (2.5, 5, and 10) hydrogels, and the CSMP hydrogel presented the largest swelling ratio. As ZnO levels rose, the CSMP-ZnO group’s swelling rate reduced, suggesting that CSMP-ZnO with higher ZnO concentrations exhibited superior anti-swelling effects.

### 3.5 *In vitro* immersion degradation behavior of hydrogels

The biodegradability and degradation behaviors of biomaterials are significant for tissue regeneration. Ideal bone repair materials should be fully biodegradable, and the degradation rate should match the bone tissue regeneration rate (48). Therefore, the *in vitro* immersion degradation behavior of the hydrogels was investigated (Figure 1h). The experiment demonstrated the degradation rate of hydrogels when they were immersed in PBS under agitation at 37 °C for 28 days, with periodic measurements taken. As the immersion time increased, the mass of all hydrogels decreased. After 28 days, the CSMP-ZnO hydrogels presented a larger mass decrease than CSMP. As the content of ZnO increasesd, the degradation ratio of ZnO hydrogel increased. Our study found that CSMP-ZnO (10) had the highest degradation rate. We speculate that the mass loss of CSMP-ZnO hydrogels might be attributed to the degradation of ZnO.

Based on the release of Zn ions in Figure 1i, these ions were largely released in the initial stage for all ZnO-incorporated CSMP hydrogels; with increasing immersion time, the Zn ion release rate decreased. As expected, more ZnO-incorporated hydrogels released more Zn ions during the immersion period. The release of Zn ions in CSMP-ZnO (10) hydrogel presented the largest Zn ion release.

### 3.6 The proliferation and viability of the rBMSCs and HUVECs

**Figure 2:**
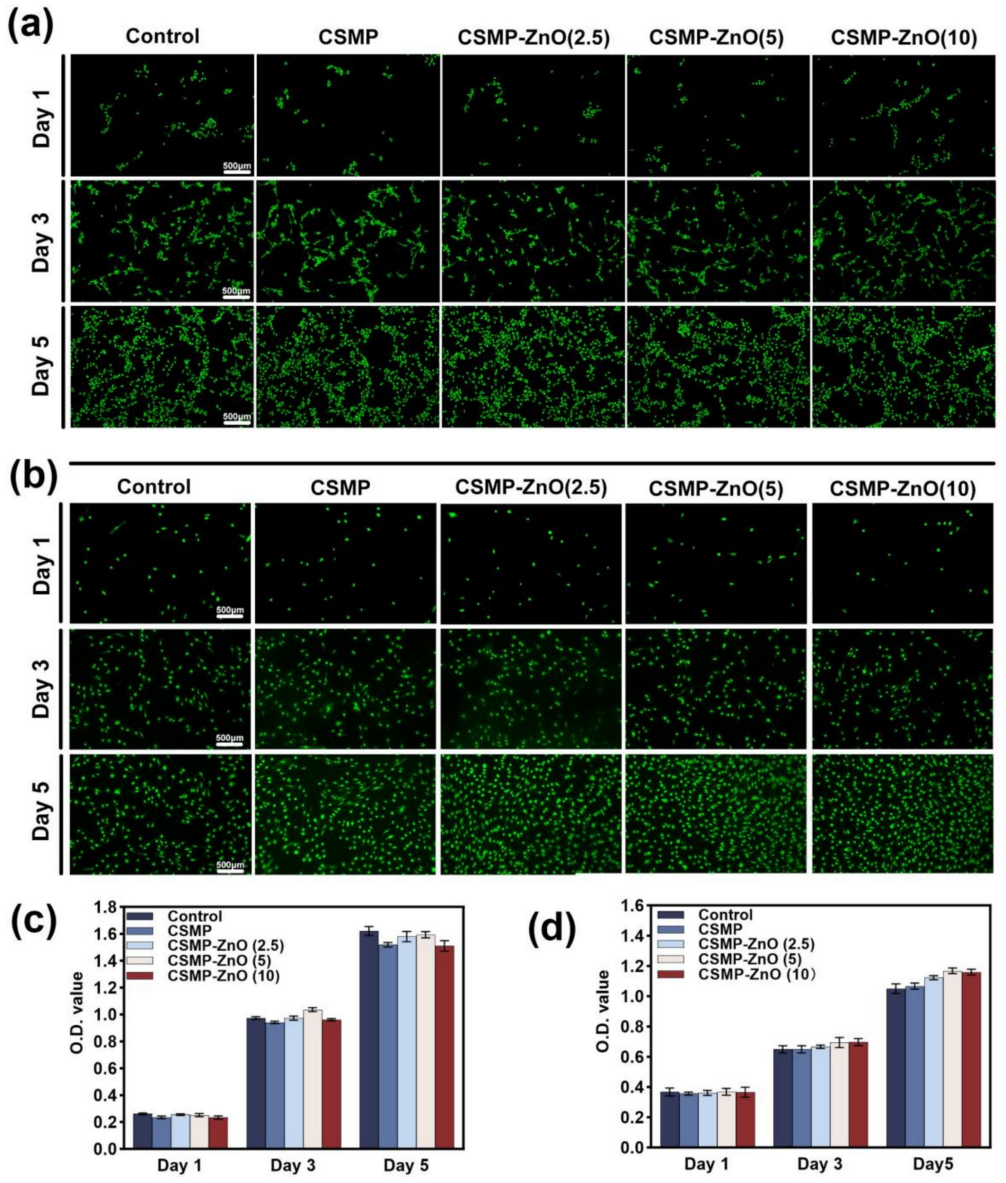
The biocompatibility of hydrogels for rBMSCs evaluated by **(a)** Calcein-AM/PI (live/dead) staining and **(c)** CCK-8 assay. The biocompatibility of hydrogels for HUVECs evaluated by **(b)** Calcein-AM/PI (live/dead) staining and **(d)** CCK-8 assay. Data are presented as mean values ± SD (*n* = 3). *Compared with control. #Compared with CSMP. *p < 0.05, **p < 0.01, ***p < 0.001.

Calcein-AM/PI (live/dead) staining was performed to explore the biocompatibility of the hydrogels by detecting the viability of co-culture rBMSCs and HUVECs at day 1 3, and 5, in which living cells were stained with green while dead cells were stained with red. All hydrogels showed excellent biocompatibility for both rBMSCs and HUVECs, as hardly any dead cells could be seen in fluorescence micrographs (Figures 2a,b). Moreover, enhanced HUVECs proliferation was observed in the hydrogels containing Zn ions (Figure 2b). The excellent biocompatibility of these hydrogels was also confirmed by quantitative analysis of CCK-8 assay (Figures 2c,d). It was found that higher OD values of HUVECs was observed in CSMP-ZnO (2.5, 5, 10) group on days 3, and 5 compared with the CSMP group, indicating that the introduced Zn ions possess the ability to promote the proliferation of HUVECs.

### 3.7 *In vitro* osteogenic propertoes of hydrogel scaffolds

**Figure 3:**
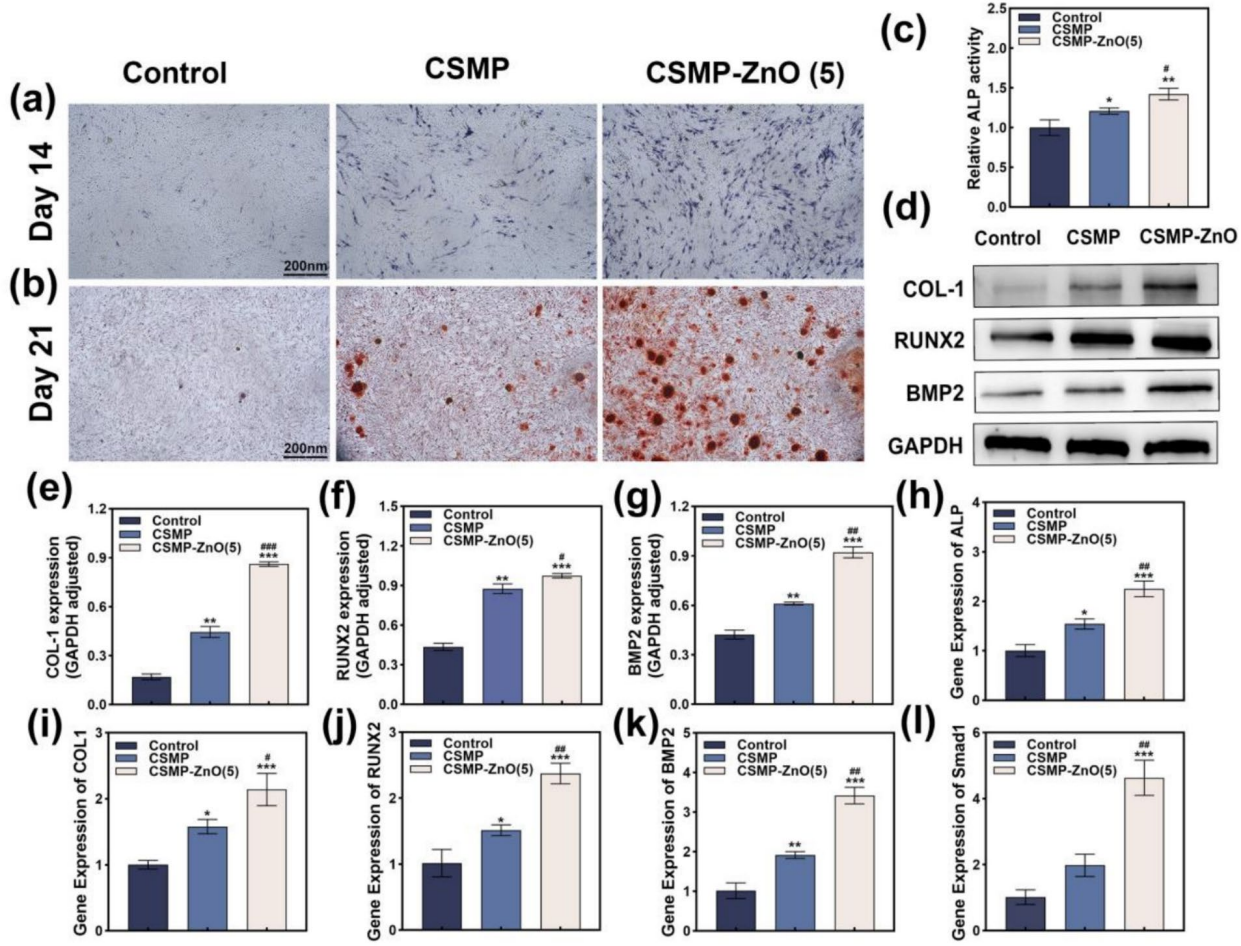
The osteogenic properties of hydrogels. **(a)** Alkaline phosphatase and **(b)** Alizarin red S staining of rBMSCs cultured with blank control, CSMP and CSMP-ZnO (5) scaffolds extration medium. **(c)** Quantitative analysis results of Alkaline phosphatase. **(d–g)** Osteogenic proteins (COL-1, RUNX2, BMP2) expression of rBMSCs cultured with blank control, CSMP and CSMP-ZnO (5) scaffolds extration medium evaluated by Western blot. **(h–l)** Osteogenic gene (COL-1, RUNX2, BMP2, Smad1, ALP) expression of rBMSCs cultured with blank control, CSMP and CSMP-ZnO (5) scaffolds extration medium evaluated by qPCR. Quantitative analysis results of osteogenic proteins expression of rBMSCs using ImageJ. Data are presented as mean values ± SD (*n* = 3). *Compared with control. #Compared with CSMP. *p < 0.05, **p < 0.01, ***p < 0.001; ^#^*p* < 0.05, ^##^*p* < 0.01, ^###^*p* < 0.001.

To further verify the bone-inducing and mineralizing abilities of each group of scaffolds, *in vitro*, ALP staining, ALP activity assay, and ARS staining were performed. From the ALP staining images (Figure 3a), we observed that on day 14, all the hydrogel samples’ staining became dark. Among the composite hydrogels, CSMP-ZnO (5) exhibited an intensely dark color. The ALP activity is represented in (Figure 3c). Compared to other groups, the cells in the CSMP-ZnO (5) scaffolds showed the highest activity. The CSMP scaffold group had higher activity than the control group but was less than that of the CSMP-ZnO (5) scaffold group. To identify and estimate the formation of calcium nodules in the ECM of rBMSCs after exposure to all the hydrogels for 21 days. ARS demonstrated similar results to ALP staining and activity (Figure 3b). CSMP-ZnO (5) hydrogels showed a larger and deeper red area than other hydrogels. The CSMP-ZnO (5) hydrogel was chosen for the subsequent experiments for the following reasons: (1) CSMP-ZnO (5) exhibits excellent rheological properties, compressive properties, anti-swelling effects, and degradation; (2) CSMP-ZnO (5) demonstrates favorable biocompatibility.

**Figure 4:**
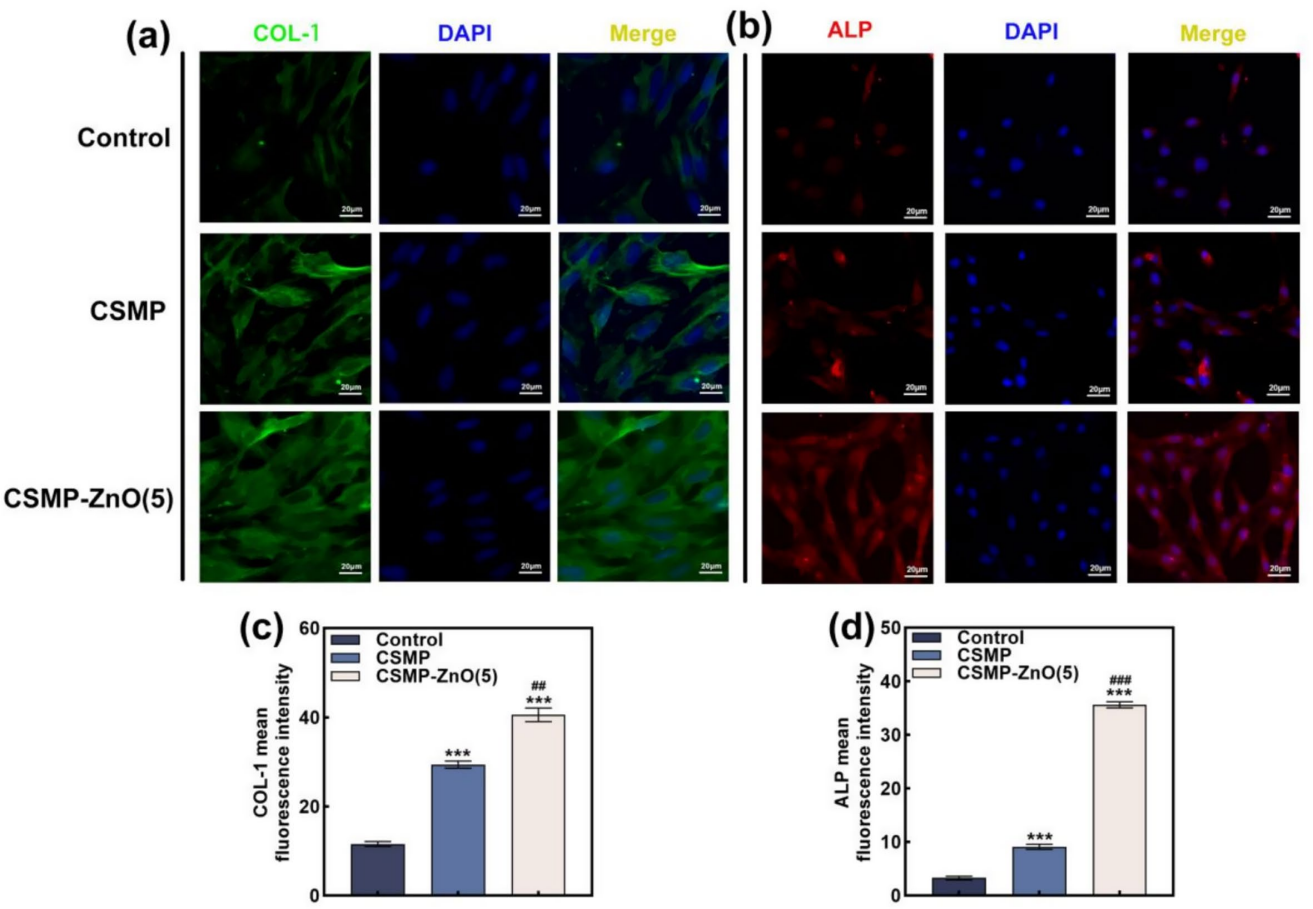
Representative immunofluorescence images of rBMSCs cultured with blank control, CSMP and CSMP-ZnO (5) scaffolds extration medium: **(a)** COL-1 and **(b)** ALP. **(c,d)** Quantitative analysis of IF for COL-1 and ALP. Data are presented as mean values ± SD (*n* = 3). *Compared with control. #Compared with CSMP. *p < 0.05, **p < 0.01, ***p < 0.001; ^#^*p* < 0.05, ^##^*p* < 0.01, ^###^*p* < 0.001.

It was observed that CSMP-ZnO scaffolds can promote osteoblast differentiation. Hence, we further explored the molecular mechanism of the above phenomenon. By employing WB and qRT-PCR, the osteoblast-specific proteins (Figures 3d–g) and genes (Figures 3h–l) were detected. Compared to the control group, the osteoblast-related proteins (COL-1, RUNX2, and BMP2) were higher in the CSMP and CSMP-ZnO. Similarly, the gene expression (*COL-1*, *RUNX2*, *BMP2*, *Smad1*, and *ALP*) in the CSMP and CSMP-ZnO groups was also higher, with the CSMP-ZnO group showing superior osteogenic properties. COL-1 is the major component of the organic matrix of bone, which promotes calcium and phosphorus deposition and accelerates new bone tissue calcification (49). *RUNX2* is a fundamental transcription factor for bone development and regulating the differentiation of mesenchymal stem cells into osteoblasts. In immature osteoblasts, *RUNX2* regulates the expression of bone matrix protein genes, including COLA1 and COLA2, and induces osteoblast maturation (50). BMP2 and Smad1 are target proteins in the classic osteoblast differentiation pathway, stimulating the mesenchymal stem cells to differentiate into osteoblasts (51). We speculated that the Zn^2+^ ions from the CSMP-ZnO scaffolds promoted osteoblast differentiation by activating the BMP2–Smad1 signaling pathway. Regarding osteoinductive genes and proteins, the osteoinductive ability isthe control < CSMP < CSMP-ZnO. Simultaneously, the CSMP-ZnO scaffold exhibited a more pronounced impact on enhancing osteoblast differentiation, possibly attributable to the synergistic influence of Zn^2+^. In addition, the protein levels of COL-1 and ALP were visualized using a fluorescence microscope. The immunofluorescence images showed the augmented expressions of COL-1 (Figures 4a,c) and ALP (Figures 4b,d) in the CSMP-ZnO group compared to those in the CSMP hydrogel and control groups.

### 3.8 *In vitro* angiogenic properties of hydrogel scaffolds

**Figure 5:**
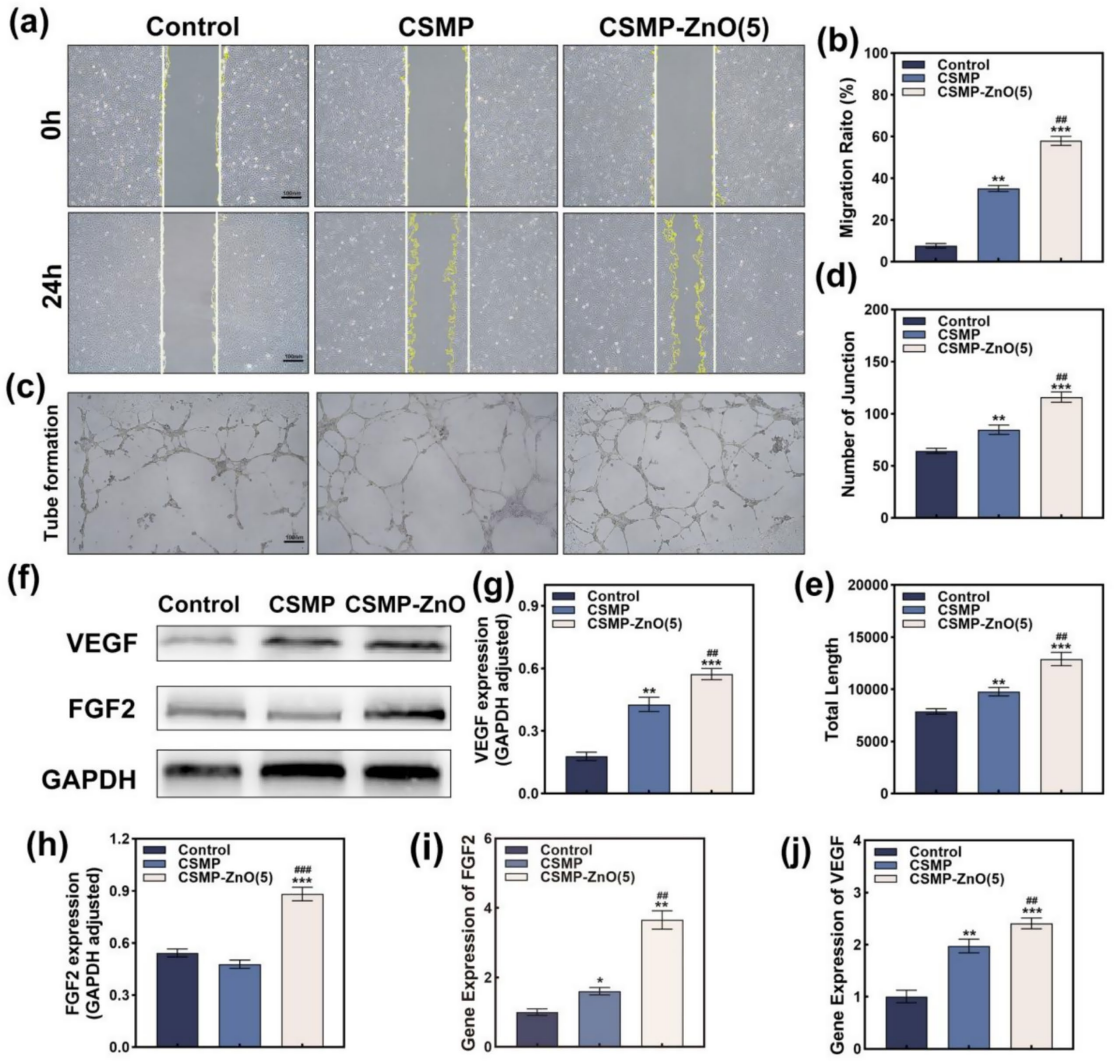
**(a)** Scratch assay and **(b)** corresponding quantitative analysis evaluating the migration activity of HUVECs. **(c)** Optical microscope images of Matrigel experiment evaluating the tube formation ability of HUVECs. **(d,e)** Quantitative analysis of the number of joint and total length. **(f–h)** Angiogenic protein (VEGF and FGF2) expression of HUVECs evaluated by WB. **(i,j)** Angiogenic gene (*VEGF*, *FGF2*) expression of HUVECs evaluated by PCR. Data are presented as mean values ± SD (*n* = 3). *Compared with control. #Compared with CSMP. *p < 0.05, **p < 0.01, ***p < 0.001; ^#^*p* < 0.05, ^##^*p* < 0.01, ^###^*p* < 0.001.

To determine the *in vitro* angiogenic properties of the hydrogels, scratch assay, tanswell assay, and tube formation assay were performed using HUVECs. As shown in Figure 5a, scratches with the same width were made on the bottom of each well at 0 h. After 24 h healing, significant thinner scratches were observed in CSMP-ZnO groups while much wider scratches remained in the Control and CSMP groups, suggesting that the addition of ZnO in hydrogels could significantly accelerate cell migration. Further quantitative analysis showed that the migration ratio of HUVECs in CSMP-ZnO group was 1.6-fold higher than that in CSMP group (Figure 5b).

Another transwell experiment using crystal violet staining to characterize permeabilized cells was performed to confirm the effect of hydrogels on cell migration. As presented in Supplementary Figure S4, significantly more migrated cells were observed in the CSMP-ZnO groups compared with CSMP and control groups. Therefore, it suggests that the ions released could promote cell migration.

To further verify the angiogenesis properties of differnet hydrogels, the matrigel experiment (tube formation assay) that can directly reflect the vascularity generation level was conducted. In Figure 5c, significantly more tube structures were observed in CSMP-ZnO groups. Further quantitative results demonstrated 1.37-fold and 1.32-fold increase of junction number and total length in CSMP-ZnO group compared with CSMP group (Figures 5d,e). Moreover, the expresion of angiogenic genes (*VEGF* and *FGF2*) and protiens (VEGF and FGF2) of HUVECs cultured with different hydrogels was detected. Similarly, significantly higher angiogenic genes (1.22-fold for *VEGF* and 2.28-fold for *FGF2*) (Figures 5f,g) and protiens expression (1.85-fold for VEGF and 1.34-fold for FGF2) (Figures 5h–j) were observed in CSMP-ZnO and CSMP groups, indicating their better angiogenic properties. In conclusion, the CSMP-ZnO hydrogel could accelerate cell migration, activate angiogenic gene expression, and thus promote angiogenesis performance of hydrogels.

### 3.9 Osteogenic activities of the hydrogel scaffolds *in vivo*

**Figure 6:**
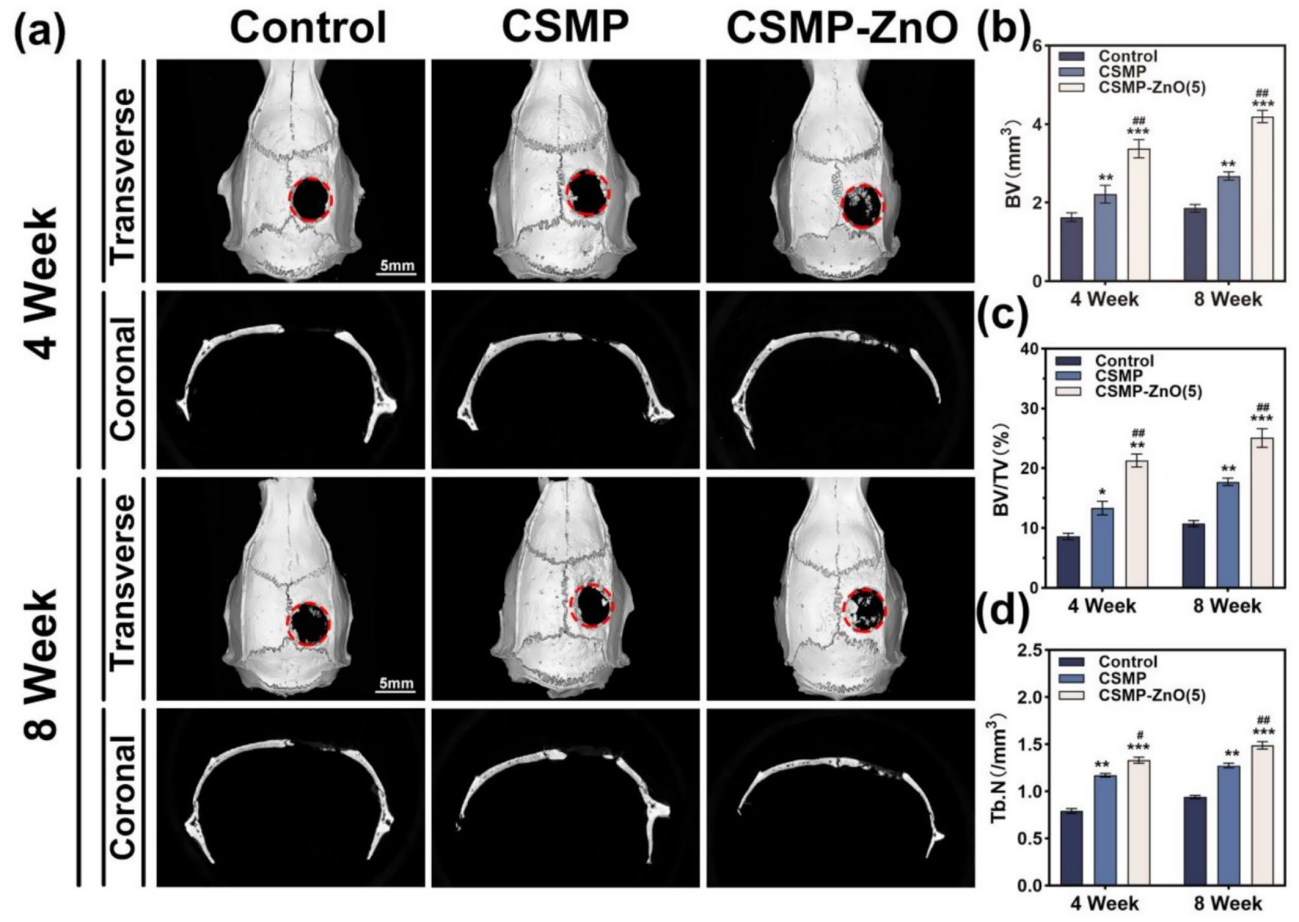
Micro-CT of new regenerated bone for the hydrogels implanted in the SD rat 5 mm critical-sized cranial defect for 4 and 8 weeks. **(a)** Micro-CT images. **(b–d)** New bone volume (BV), New bone volume/tissue volume (BV/TV) ratios and Trabecular bone number (Tb.N) in the critical-sized area calculated from micro-CT assessment. *Compared with control. #Compared with CSMP. Data are presented as mean values ± SD (*n* = 3). *p < 0.05, **p < 0.01, ***p < 0.001; ^#^*p* < 0.05, ^##^*p* < 0.01, ^###^*p* < 0.001.

The hydrogels were implanted into the 5 mm rat cranial defects to investigate their *in vivo* osteogenic properties. Following 4 and 8 weeks of implantation, the cranial bone was collected for micro-CT analysis to evaluate the newly formed bone. Based on the micro-CT results from (Figure 6a), a larger area of regenerated bone was observed for the CSMP-ZnO (5) hydrogels at 4 weeks than the CSMP and control grouops. As the implantation period increased, the regenerated bone area increased for CSMP-ZnO (5) hydrogels, but the control group presented no significant increase. The statistical analysis results for BV, BV/TV, and Tb.N are shown (Figures 6b–d). At 4 weeks, the BV and BV/TV of regenerated bone for CSMP-ZnO (5) were higher than the CSMP and control groups, and Tb. N also showed a similar tendency. The Tb. N of regenerated bone for CSMP-ZnO (5) hydrogel was higher than in other groups. Over time, this tendency remained the same.

**Figure 7:**
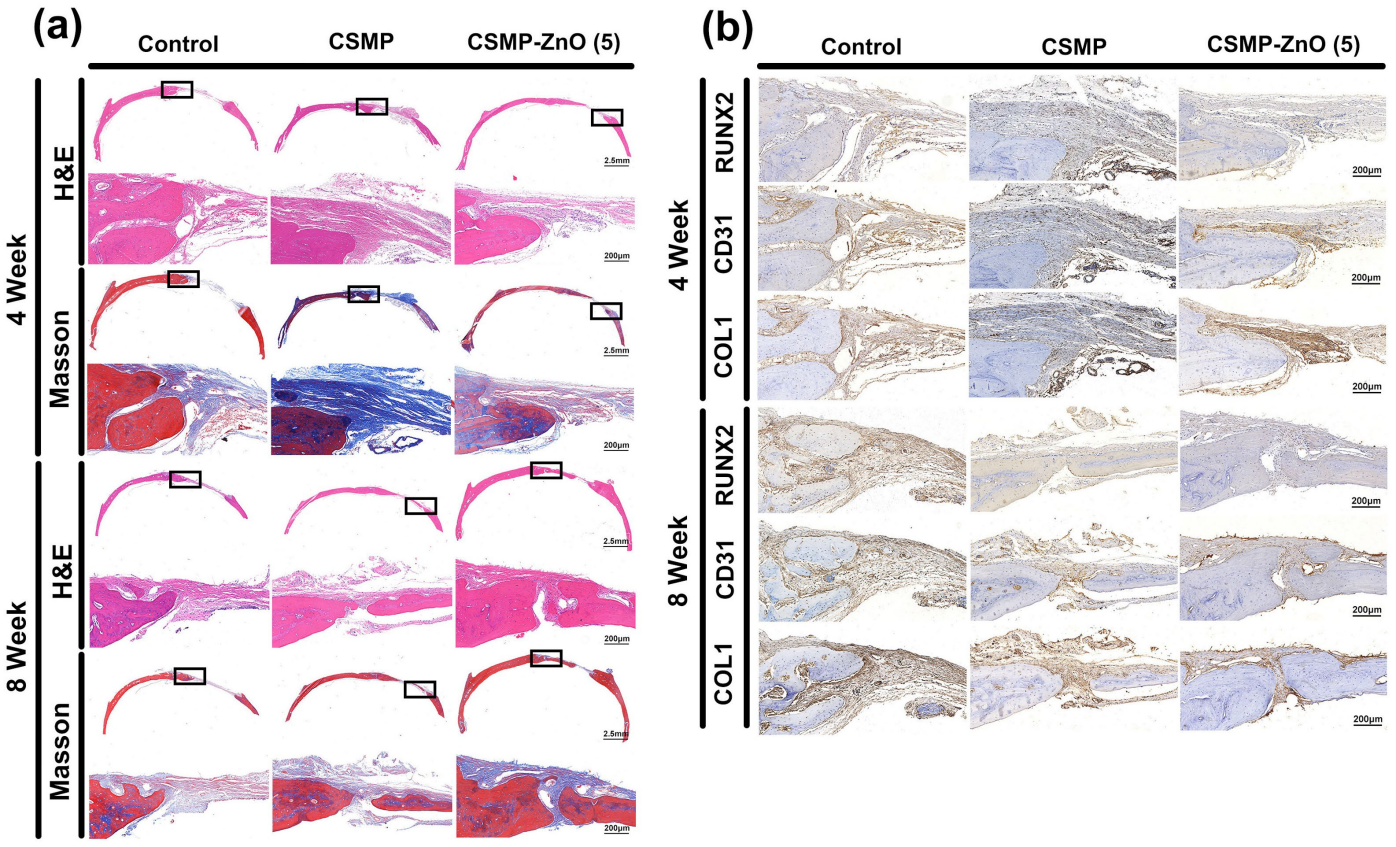
Section staining for the critical-sized cranial defect area after hydrogels implanted for 4 and 8 weeks. **(a)** Representative hematoxylin and eosin (H&E) and Masson trichrome stained images for the defect area after hydrogels implanted for 4 and 8 weeks. **(b)** Immunohistochemical staining of the osteogenic marker RUNX2, COL-1 and CD31.

H&E and Masson’s trichrome staining further verified the histological formation and maturity of new bone (Figure 7a). The H&E staining revealed that the CSMP-ZnO (5) hydrogel group exhibited significant new bone and blood vessel repair at the bone defect site, with no remaining undegraded implantation. As shown in Supplementary Figure S5, Supporting Information, no difference was observed in the heart liver, spleen, kidney and lung tissue sections between 0-week, 4-week, 8-week after sample implantation, confirming the excellent biocompatibility of CSMP-ZnO. Significantly, the growth of new blood vessels was also seen in the CSMP-ZnO (5) group. After 4 weeks of implantation, the stained area was larger for CSMP-ZnO (5) than CSMP. When the implantation period increased, the stained areas for all hydrogels increased significantly for CSMP-ZnO (5) hydrogels. The CSMP-ZnO (5) implanted area presented more new bone area than the other two groups. After 8 weeks of implantation, in the CSMP-ZnO (5) group, the new bone regeneration fully repaired the bone defect site, generation mature bone tissue and vascularization. In the regenerated tissue, there was superior columnar organization and integration compared to other groups. Furthermore, the CSMP-ZnO (5) hydrogel contains abundant collagen fibers. Compared to the CSMP hydrogel, more new cells, dense collagen, and new vasculature were observed at the center of the bone defect site in the CSMP-ZnO (5) hydrogel group. These results suggest that CSMP-ZnO (5) hydrogel promotes osteogenesis and angiogenesis and inhabits fibrous tissuec growth.

Masson’s staining was used to analyze the collagen and newly formed bone. Masson’s trichrome staining shows that mature bone matrix (collagenous tissue) is stained blue, and muscle tissues and red blood cells are also stained red. Only a small amount of new, immature bone matrix (mainly including muscle fibers and partially collagen) can be observed in the control group. In contrast, the newly formed collagen (blue) and blood vessels (red) were obvious in CSMP and CSMP-ZnO (5) hydrogels after 4 weeks, similar to the results of H&E staining. CSMP-ZnO (5) hydrogel group has abundant and denser collagen and blood vessels. The control group exhibited a limited amount of mature bone and newly formed blood vessels at the defect site after 8 weeks. In contrast, the CSMP-ZnO (5) group exhibited a substantial amount of mature bone matrix and a greater number of blood vessels. The well-arranged collagens were stained a deep blue color, and more blood vessels were growing at the bone defect site, indicating that CSMP-ZnO (5) hydrogel can osteogenesis and angiogenesis. H&E and Masson’s trichrome staining showed no inflammatory response or necrosis in the newly formed fibrous tissue in all the groups. However, dense, mature, freshly formed fibrous tissue and neovascularization were observed in the CSMP-ZnO (5) group, suggesting that blood supply can promote fibrous tissue fromation.

The results were confirmed by immunohistochemical staining of RUNX2, COL-1 and CD31 (Figure 7b). The findings demonstrated a significant increase in immunofluorescence (RUNX2 and COL-1) in the CSMP-ZnO (5) group, with a well-organized distribution around the osteoblasts. This suggests that the controlled release of zinc may enhance the expression of RUNX2 and COL-1, thereby stimulating specific biological processes. Furthermore, the positive staining of CD31 was significantly improved in the CSMP-ZnO (5) hydrogel group compared to other groups. Therefore, the CSMP-ZnO (5) hydrogel could significantly promote bone and vascular regeneration.

## 4 Discussion

In this study, we followed Chen et al.’s method to prepare CSMP [15]. First, methacrylic anhydride reacted with CS to obtain CSMA. The CSMA solution was directly added to the PS mix solution in the second phosphate step. Subsequently, we added ZnO-NPs to the CSMP solution, and the phosphate groups on CSMP bound with Zn^2+^ through chelation. In this hydrogel, the PS was grafted onto CS to provide phosphate groups to chelate with ZnO-NPs. SEM images showed that the porous structure of CSMP hydrogel, with pore sizes ranging from 50 to 100 μm, is more suitable for cell and tissure growth and nutrient and metabolite transport, waste removal, and oxygen diffusion (52). For bone graft materials, mechanical properties, like compressive strength, play a crucial role in bone regeneration (53). In addition, with an increase in ZnO-NPs, the maximum compressive strength of CSMP-ZnO rose, possibly due to ZnO-NPs acting as cross-linkers in this hydrogel system. Thus, with more ZnO-NPs in the hydrogel, the cross-linking sites and degree were increased, improving the compression property (54). Due to the weakly acidic nature of the CSMP solution, a small amount of ZnO was dissolved during the formation of the CSMP-ZnO hydrogel. However, when most of the phosphate groups were bound to ZnO, the dissolution of ZnO will stopped. Throughout the experiment, it was found that the cross-linking degree of CSMP-ZnO (5) hydrogel was higher, resulting in better mechanical properties. However, the compressive strength of CSMP-ZnO (10) was reduced, possibly because some ZnO-NPs did not combine with the hydrogel. When ZnO NPs were added at a concentration of 10 mg/mL, the maximum compressive strength and modulus decreased. This may be because ZnO-NPs are not hard-phase structures.

For bone repair materials, excellent biocompatibility means that the implants can promote the development of tissues and cells in the bone defects environment, such as the proliferation of osteogenic-related cells and the differentiation of osteocytes. The application of ZnO-NPs *in vivo* is also considered safe. Our results indicate that CSMP and CSMP-ZnO hydrogels did not inhibit cell proliferation and have good cell adhesion properties. The rate of bone regeneration is significantly influenced by the impact of implants on cellular osteogenic differentiation, and the nature of bone as a mineralized connective tissue depends on the function and interaction of the cells with the extracellular matrix. The functional role of osteoblasts in bone formation is divided into three main stages. The first stage is the adhesion and proliferation of osteoblasts, and previous experiments have demonstrated that CSMP-ZnO hydrogel can promote the proliferation and adhesion of rBMSCs osteogenic precursor cells. The second stage is osteogenic differentiation, which is the process of differentiation and maturation of osteoblasts from osteogenic precursor cells. In the third stage, the extracellular matrix mineralization, fully developed osteoblasts create a bone matrix by depositing calcium and phosphorus (55, 56). In this study, the CSMP-ZnO hydrogel promoted the expression of *COL1*, *RUNX2*, *BMP2*, *Smad1,* and *ALP* and the expression of COL1, RUNX2, and BMP2 proteins in rBMSCs. Zinc can influence bone formation in the human body through various pathways. The results have indicated that zinc can enhance bone formation by regulating osteoblast-specific transcription factor 2 expression, increasing osteocalcin synthesis, type I collagen, and alkaline phosphatase activity, as well as increasing the accumulation of calcium and phosphorus. Zn^2+^ effectively enhances the absorption and utilization of crucial minerals like calcium and phosphorus while activating matrix metalloproteinases. This promotes bone metabolic balance and strength, ultimately leading to an increase in bone density. These factors collectively play a pivotal role in osteogenesis and significantly contribute to new bone growth (57–59). ALP is a typical protein product produced during osteoblast proliferation, differentiation, and extracellular matrix maturation; therefore, ALP activity is often an indicator of early osteoblast differentiation (60). ALP staining showed that CSMP-ZnO hydrogel could significantly fasten cell differentiation and maturation. The calcium nodule staining results indicated a significant advancement when extracellular matrix mineralization occurred in the CSMP-ZnO hydrogel. To evaluate the bone repair capability and bioactivity of CSMP-ZnO hydrogel *in vivo*, we implanted it into the skull of rats. We analyzed the differences in bone growth between CSMP and CSMP-ZnO at various time points post-implantation. Micro-CT analysis indicates that the CSMP-ZnO hydrogel group exhibited greater new bone volume and a higher number of trabeculae than the other two groups. Observations from H&E and Masson’s staining revealed that CSMP-ZnO hydrogel can promote the formation of collagen, blood vessels, and bone matrix at the bone defect site. *COL1* and *ALP* immunohistochemical staining also confirmed this result. Compared to CSMP hydrogel and control groups, the CSMP-ZnO hydrogel group showed a significant increase in positive staining for new bone and osteoblasts, suggesting that CSMP-ZnO hydrogel can markedly promote bone regeneration and bone defect repair, and exhibit positive effects on osteogenesis and new bone formation.

Timely and adequate angiogenesis during bone defect repair is also crucial to the speed of bone repair. Neovascularization not only provides a large amount of oxygen and nutrients, but also a constant supply of bone progenitor cells and bone units. In this study, the effects of ZnO on HUVECs were analyzed via scratch assay, transwell assay and tube formation assay, and the results showed that the ZnO enhanced the migration ability and tube formation of HUVCEs compared with CSMP group. Moreover, the transcription levels of *VEGF* and *FGF2* were most significantly upregulated in CSMP-ZnO group, which is attributed to the release of Zn^2+^ from nano ZnO-NPs. Similar results were also obtained in WB by detecting the expression of the angiogenic-related protein.

In conclusion, the *in vivo* and *in vitro* experimental results demonstrate that the ZnO-NPs-doped phosphorylated CSMP hydrogel exhibits good bioactivity, osteogenic properties, and angiogenic properties.

## 5 Study limitations

However, the present study has limitations. First, the exploration of the osteogenic mechanism of CSMP-ZnO hydrogels in this experiment is preliminary, relying solely on simple validation from other studies. To overcome this limitation, we suggest that future studies employ genomics or proteomics methods to comprehensively investigate the osteogenic mechanisms, which will provide a deeper understanding of the underlying biological processes. Additionally, we acknowledge that the impact of immune regulation on bone formation was not investigated in our current experiment. Given the positive role of immune regulation in bone formation, we propose that future experiments should explore the influence of CSMP-ZnO on immune regulation during bone regeneration. This will not only enhance our understanding of the material’s biological effects but also potentially uncover new therapeutic targets.

## 6 Conclusion

Thus, we designed novel CSMP-ZnO hydrogel scaffolds and used them for local bone regeneration for the first time. The porous structure of CSMP-ZnO scaffolds enhanced cell adhesion and showed good binding with bone tissue to promote bone regeneration. The incorporation of ZnO nanoparticles improved the bulk modulus, compression stress, anti-swelling properties. Moreover, the CSMP-ZnO scaffold poeeseees the sustained release of zinc ions, and the release of zinc ions further promote the osteogenesis and angiogenesis performance. The CSMP-ZnO hydrogel scaffolds significantly promoted osteogenic activity through the BMP2–Smad1 signaling pathway, and increased the expression levels of osteogenic proteins (COL-1, BMP2, RUNX2) and genes (*COL-1*, *BMP2*, *RUNX2*, *Smad1*, *ALP*). In addition, *in vivo* experiments showed that the CSMP-ZnO hydrogel significantly promoted bone regeneration. The CSMP-ZnO hydrogel superior to the CSMP hydrogel in enhancing the growth, formation of bone tissue by rBMSCs, and the regeneration of bone. This study demonstrated that incorporating ZnO into hydrogels demonsteates a promising strategy for bone regeneration, thus serving as a new strategy for bone tissue engineering and regeneration in the future.

## Data Availability

The original contributions presented in the study are included in the article/Supplementary material, further inquiries can be directed to the corresponding authors.
