# Correction: Zinc oxide nanoparticle chelated phosphocreatine-grafted chitosan composite hydrogels for enhancing osteogenesis and angiogenesis in bone regeneration

**DOI:** 10.3389/fmed.2026.1852114

**Published:** 2026-06-10

**Authors:** Leidong Lian, Dingli Xu, Chaonan He, Zhe Luo, Han Yu, Botao Liu, Ke Zhou, Liangjie Lu, Kaifeng Gan

**Affiliations:** 1 The Affiliated Lihuili Hospital of Ningbo University, Ningbo, Zhejiang, China; 2 Health Science Center, Ningbo University, Ningbo, China; 3 Ningbo Institute of Innovation for Combined Medicine and Engineering, The Affiliated Lihuili Hospital of Ningbo University, Ningbo, Zhejiang, China

**Keywords:** bone regeneration, osteogenesis, phosphate-functionalized, chitosan, zinc oxide nanoparticles

There was a mistake in Figure 7 published. Incorrect images were erroneously included in the original submission. The corrected Figure 7 appears below.

**Figure 7:**
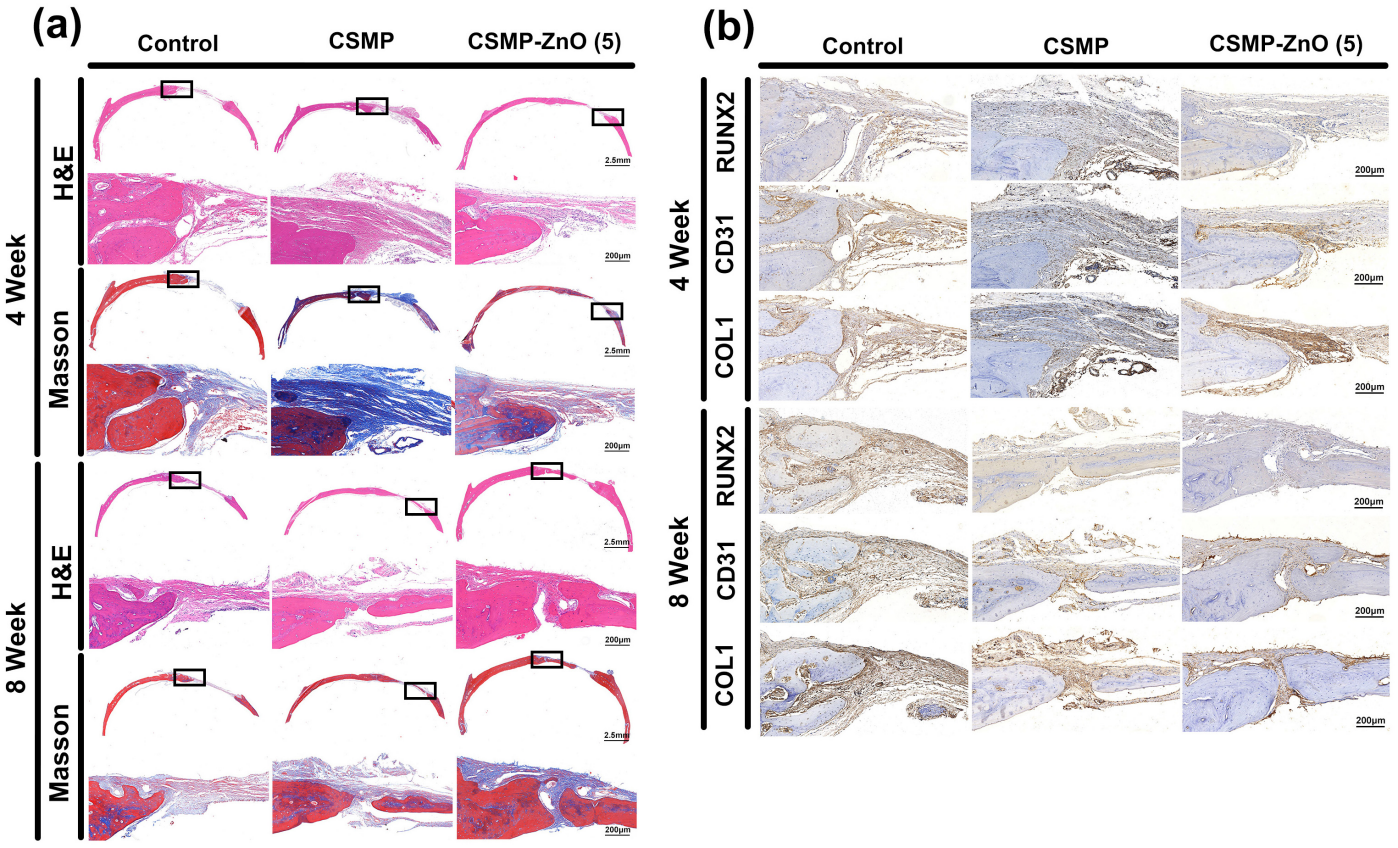
Section staining for the critical-sized cranial defect area after hydrogels implanted for 4 and 8 weeks. **(a)** Representative hematoxylin and eosin (H&E) and Masson trichrome stained images for the defect area after hydrogels implanted for 4 and 8 weeks. **(b)** Immunohistochemical staining of the osteogenic marker RUNX2, COL-1 and CD31.

The original File “Supplementary file 1” was erroneously published with the original version of this paper. The file has now been replaced with a corrected version.

The original version of this article has been updated.

