# Supplementary material for: Zinc oxide nanoparticle chelated phosphocreatine-grafted chitosan composite hydrogels for enhancing osteogenesis and angiogenesis in bone regeneration
Source: Front Med (Lausanne). 2025 Dec 5;12:1729401. doi: 10.3389/fmed.2025.1729401 (PMC12714965; doi:10.3389/fmed.2025.1729401)
Supplement: Supplementary file 1 [file Supplementary_file_1.pdf]

Supporting Imformation Available

Zinc oxide nanoparticle chelated phosphocreatine-grafted chitosan composite hydrogels for enhancing osteogenesis and angiogenesis in bone regeneration

*Leidong Lian [a, ‡], Dingli Xu [b, ‡], Chaonan He [c], Zhe Luo [a, b], Han Yu [b], Botao Liu [b], Ke Zhou [a], Liangjie Lu [a *], Kaifeng Gan [a *]*

a. The Affiliated Lihuili Hospital of Ningbo University, Ningbo, Zhejiang, 315211, People's Republic of China

b. Health Science Center, Ningbo University, Ningbo, 315100, People's Republic of China

c. Ningbo Institute of Innovation for Combined Medicine and Engineering, The Affiliated Lihuili Hospital of Ningbo University, Ningbo, Zhejiang, 315040, China

‡ These authors contributed equally to this work and should be considered co-first authors.

Corresponding Author:

Kaifeng Gan, Email: gankaifeng03@163.com;

Liangjie Lu, Email: lhlluliangjie@nbu.edu.cn

SUPPLEMENTARY FIGURES

Western blot data		
**Species**	**Protein**	**Concentration**
**Homo sapiens**	**VEGF**	**1:2000**
**Homo sapiens**	**FGF2**	**1:1000**
**Rat**	**COL-1**	**1:500**
**Rat**	**RUNX2**	**1:1000**
**Rat**	**BMP2**	**1:1000**

Figure S1. The concentrations of antibodies used in the Western blot

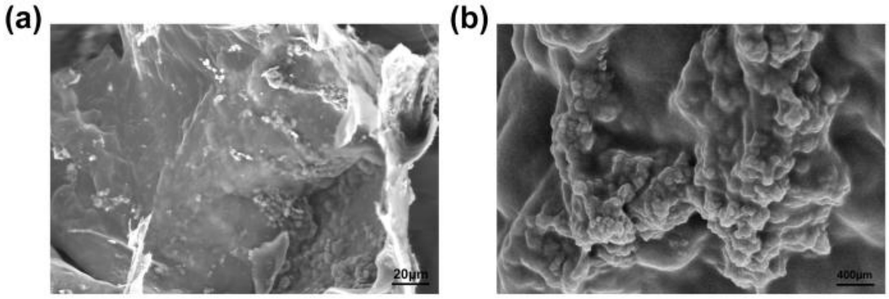

Figure S2. The SEM images of CSMP-ZnO (5): (a) 500× picture; (b) 1000× picture

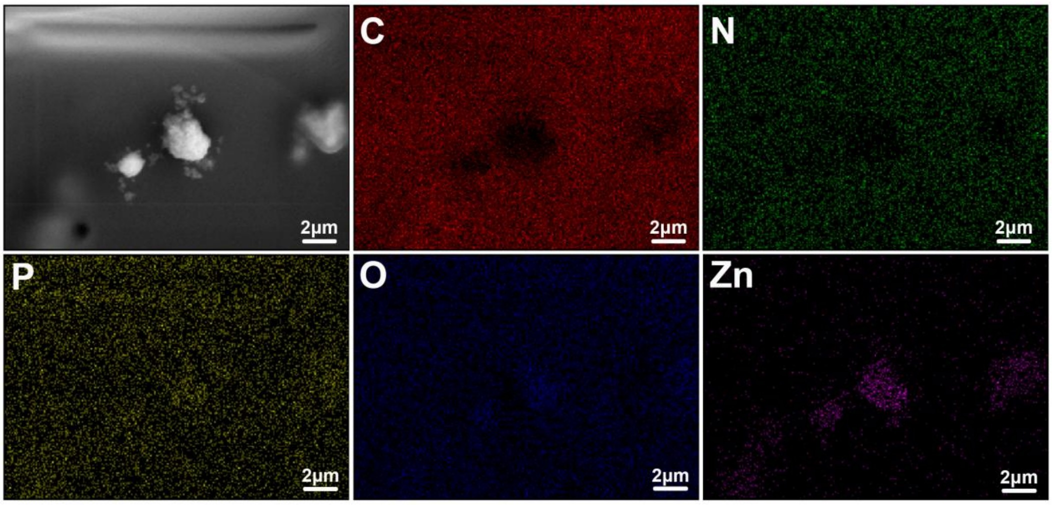

Figure S3. The surface EDS mapping results of CSMP-ZnO (5) show the distribution of elements.

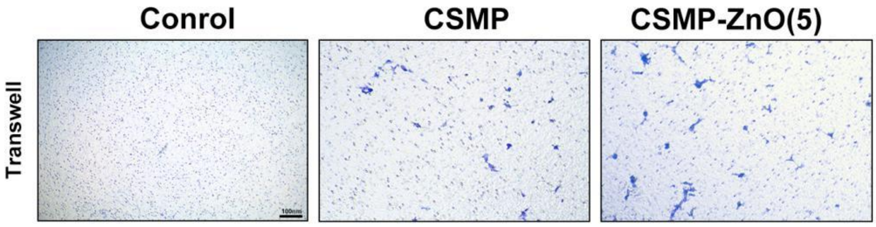

Figure S4. Transwell assay accessing the migration activity of HUVECs and the migrated cells were stained with crystal violet.

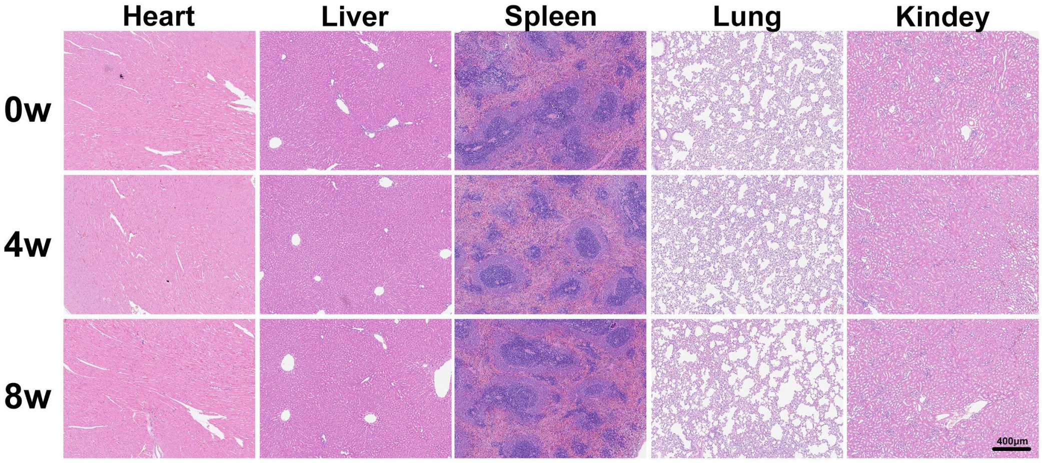

Figure S5. The tissue sections H&E staining of the heart, liver, spleen, lung, and kidney taken at 0, 4 and 8 weeks after CSMP-ZnO hydrogel implantation.
